# Intrasplenic Arterial Aneurysms during Pregnancy

**DOI:** 10.1155/2015/248141

**Published:** 2015-02-24

**Authors:** Mahmoud M. S. Abu-khalaf, Sokiyna M. Al-Ameer, Moath M. Smadi, Ayman Qatawneh, Osama A. Smara, Azmy T. Hadidy

**Affiliations:** ^1^Department of General Surgery, Jordan University Hospital and Faculty of Medicine, University of Jordan, Amman, Jordan; ^2^Department of Obstetrics & Gynecology, Jordan University Hospital and Faculty of Medicine, University of Jordan, Amman, Jordan; ^3^Department of Radiology, Jordan University Hospital and Faculty of Medicine, University of Jordan, Amman, Jordan

## Abstract

Splenic artery aneurysms account for about 60% of all visceral aneurysms. Pregnancy is a risk factor for splenic artery aneurysms rupture with high maternal mortality and fetal loss. Intrasplenic arterial aneurysms are extremely rare and have not been reported to be associated with pregnancy. This report presents a 34-year-old woman during the second trimester, admitted with severe left upper quadrant and left shoulder pain. She had two uncomplicated intrasplenic aneurysms. Splenectomy was done. She delivered a full term healthy girl. This is the first report of acute abdomen during pregnancy caused by intrasplenic artery aneurysms with maternal and fetal survival.

## 1. Introduction

Intrasplenic arterial aneurysms/pseudoaneurysms involving the intrasplenic arterial branches are very rare. They have the potential for life-threatening complications; and they must be diagnosed and treated immediately. True aneurysms inside the spleen are extremely rare. Posttraumatic intrasplenic pseudoaneurysms following nonoperative management of blunt splenic injuries are seen more frequently. The spontaneous nontraumatic pseudoaneurysms are also encountered. We report a 34-year-old woman in her second trimester, who presented with severe abdominal pain. 

## 2. Case Report

**Figure 1:**
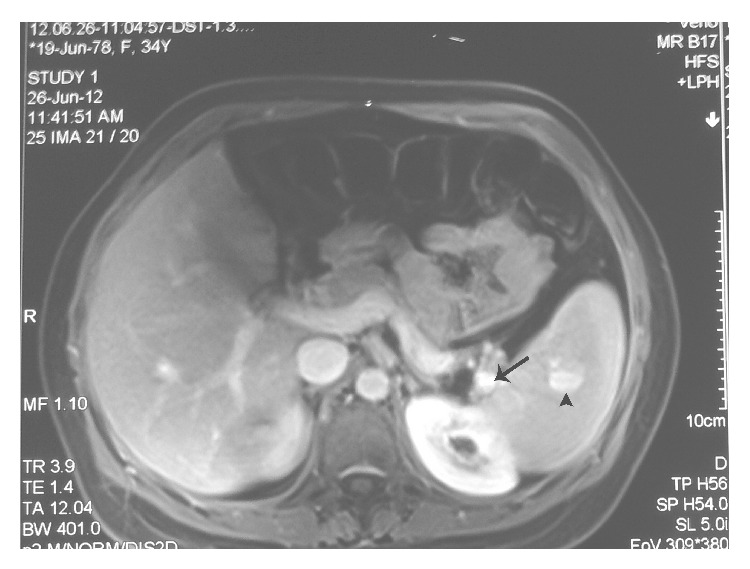
Axial T1W image with IV contrast of the upper abdomen (venous phase) showing two abnormal rounded enhancing intraparenchymal (arrow head) and hilar (arrow) splenic lesions consistent with aneurysms.

**Figure 2:**
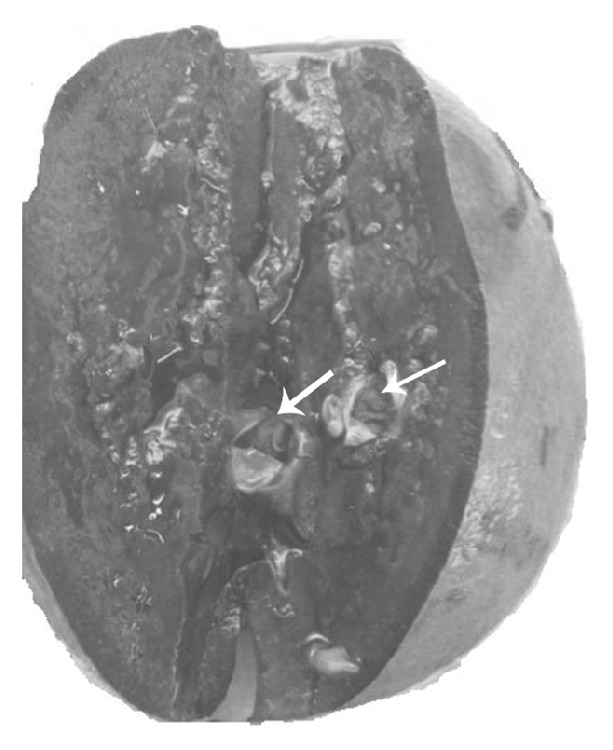
Bisected spleen showing the intrasplenic arterial aneurysm (marked by the arrow).

**Figure 3:**
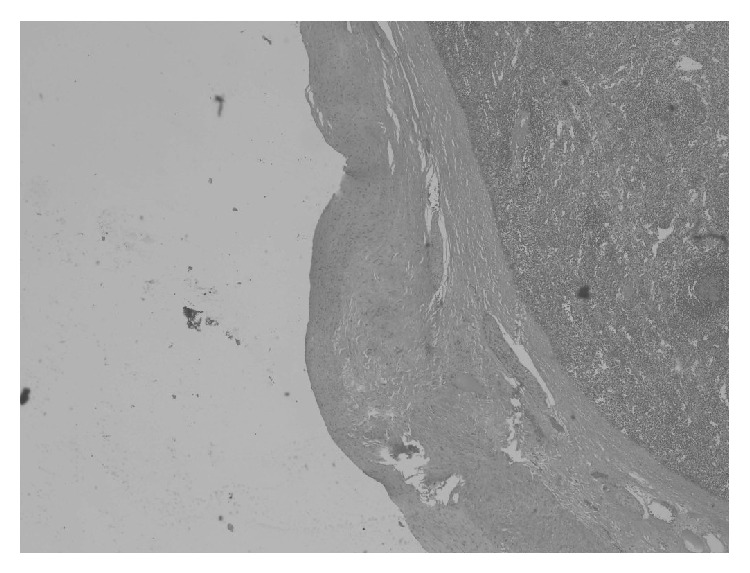
Wall of intrasplenic aneurysm showing all arterial layers. H&E 2x magnification.

A 34-year-old G8P2+5 woman presented to Jordan University Hospital on June 8, 2012 with chief complaints of left upper quadrant, left Loin, and suprapubic pain. She was diagnosed and treated as left pyelonephritis. Abdominal ultrasound (US) and colored Doppler US revealed two splenic cystic lesions: one within the splenic parenchyma (18 mm) and the other at the splenic hilum (16 mm). Both lesions showed arterial blood flow and thus were diagnosed as two splenic arterial aneurysms. The patient improved and was discharged. She was readmitted two weeks later with severe left upper quadrant and left shoulder pain. On physical examination, the patient was in good health but in severe pain. Her abdomen was tender on the left side. There was no history of either abdominal trauma or pancreatitis. She was treated for hypertension during her previous pregnancies. Her laboratory investigations including complete blood count were unremarkable. Abdominal MRI with and without intravenous contrast showed two cystic intraparenchymal and hilar splenic lesions (Figure 1). The lesions were of heterogeneous signal on T1 and T2 images with central flow void. After contrast injection, a vivid enhancement in both lesions was noted confirming the diagnosis of splenic artery aneurysms. For fear of splenic rupture with intraperitoneal haemorrhage, an urgent laparotomy was made. Splenic enlargement with no perisplenic haematoma or splenic rupture was noted. Splenic artery ligation and splenectomy were performed. Her recovery was uneventful and the platelet count increased to 864 × 10^9^/L. Cross section of the spleen disclosed two aneurysms in its lower part. One was totally intrasplenic while the other protruded into the splenic hilum (Figure 2). Both aneurysms were true ones and filled with unclotted blood (Figure 3). Her pregnancy continued and she delivered a 3.5 Kg full term healthy baby through a cesarean section.

## 3. Discussion

Splenic artery aneurysm is the third most common intra-abdominal aneurysm. It is also the commonest visceral artery aneurysm [1–3]. Most aneurysms develop in the main trunk. Aneurysms distal to the primary bifurcation are uncommon and occasionally involve small branches at the hilum [4]. Intrasplenic arterial aneurysms (ISAAs) are rare. Medline search revealed 27 publications with no previous reports of ISAAs during pregnancy, labour or postpartum period found. This is the first report of multiple ISAAs during pregnancy presenting with an acute abdomen, treated by splenectomy, with survival of the mother and fetus.

Intrasplenic arterial aneurysms (ISAAs) are either true or pseudoaneurysms, the latter being a much more common variety. Our patient had two true aneurysms: one was totally intrasplenic while the second was intrasplenic aneurysm that protruded into the hilum of the spleen.

The true ISAAs develop in patients with liver cirrhosis, portal hypertension [5, 6], infective endocarditis [7, 8], vascular malformation [9, 10], and Wilson's disease [11]. True ISAAs may be single or multiple and may be associated with extrasplenic artery aneurysms [5] or other vascular aneurysms [12].

Intrasplenic pseudoaneurysms (ISPs) result from destruction of the full thickness of the arterial wall. They are either posttraumatic (TISPs) or nontraumatic (NTISPs). The nonsurgical management of blunt splenic trauma gained popularity during the last two decades. TISPs have been reported more frequently, following blunt trauma [13–16], stab wound [17], iatrogenic interventional radiology, or biopsy procedures [18].

Nontraumatic intrasplenic pseudoaneurysms (NTISPs) are rare complications of splenic infarction, infiltration by malignant systemic disorders, infectious process, chronic pancreatitis, and arteritis [19, 20]; they are known to cause spontaneous nontraumatic splenic rupture with hemoperitoneum and they can be single or multiple, are more common in men, and are extremely fragile.

ISAAs are usually not calcified and are typically unrelated to atherosclerotic disease, while extrasplenic aneurysms often calcify and are caused by atherosclerosis.

Most uncomplicated aneurysms are likely to be asymptomatic. However, left upper quadrant or epigastric pain may occur in patients with intact splenic artery aneurysms. In recent reviews, nearly half of patients with aneurysms complained of abdominal pain. Intact ISAAs may produce pain. Our patient did have pain in the left upper abdomen and left flank for over two weeks prior to admission. The abdominal pain in our patient could be multifactorial. She had splenic enlargement and her aneurysms could have been expanding. Also, the rising estrogen may have contributed to deterioration of her pain. In addition, the hypertension during her pregnancy could have been a factor in the pathogenesis of her aneurysm.

Most patients with significant pain are experiencing rupture or acute aneurysmal expansions. Patients with ISAAs expand and then rupture into splenic parenchyma in their way to the peritoneal cavity, causing acute left upper abdominal, left shoulder pain, shock, and abdominal distension. Death can potentially result. Patients with TISPs or NTISPs usually present with intrasplenic, subcapsular, perisplenic, or intraperitoneal rupture [14]. In case they develop as a complication of nonoperative management of splenic trauma, they can be diagnosed during follow-up prior to rupture with the use of US, colour Doppler US, or CT scan screening [13]. NTISPs may present with splenic rupture or with splenic infarction as a result of thrombosis of the pseudoaneurysms [19].

Intrasplenic aneurysms and pseudoaneurysms are initially investigated by US which usually show anechoic lesion in the spleen. It may also identify haematoma in and around the spleen in cases of TISPS [17]. Colour Doppler US demonstrates turbulent flow within the lesion [17]. The use of ultrasound contrast agent clearly depicts the aneurysm/pseudoaneurysm and the arterial feeder vessel simultaneously enhancing the splenic artery [17]. Superselective angiography will finally confirm the diagnosis and the indication for endovascular therapy [17]. In the arterial phase of contrast-enhanced CT angiography, the aneurysms/pseudoaneurysms enhances simultaneously with the splenic artery and may show the intrasplenic arterial branch leading to the aneurysms/pseudoaneurysms [17]. MRI may be the least invasive imaging in a pregnant patient [15]. In our patient we made the diagnosis and confirmed it using US, colour Doppler, and MRI.

ISAAs potentially expand and cause delayed rupture of the spleen. Thus, early diagnosis and treatment are crucial [17]. If diagnosed prior to rupture, splenic embolization may be the standard of care. We did not consider angioembolization in our patient because the skillfulness of interventional angiography is not well developed in our institution for use in pregnant patients.

Delayed splenic rupture secondary to intrasplenic aneurysms/pseudoaneurysms requires laparotomy followed by splenectomy [20] and only exceptionally by splenorrhaphy [13]. We came across no attempt to use laparoscopic splenectomy for the management of such cases. This could be explained by the rarity of the event.

NTISPs present with spontaneous splenic rupture or with splenic infarction secondary to aneurysmal or pseudoaneurysmal thrombosis. The vast majority requires splenectomy [19]. We elected to treat our patient by splenectomy though partial splenectomy was technically feasible.
